# First Non‐Invasive Monitoring of Fecal Steroids in Greater Caribbean Manatees (*Trichechus manatus manatus*)

**DOI:** 10.1002/zoo.70070

**Published:** 2026-04-25

**Authors:** Vanessa Bermúdez‐Cardona, Roberto Sánchez‐Okrucky, José A. Sandoval‐Zárate, Nataly Castelblanco‐Martínez

**Affiliations:** ^1^ Facultad de Ciencias Exactas y Naturales Universidad de Buenos Aires Buenos Aires Argentina; ^2^ Asociación de Especialistas en Bienestar Animal Playa del Carmen México; ^3^ Dirección General de Zoológicos y Conservación de la Fauna Silvestre SEDEMA‐GCDMX Ciudad de México México; ^4^ Departamento de Reproducción, Facultad de Medicina Veterinaria y Zootecnia Universidad Nacional Autónoma de México Ciudad de México México; ^5^ Laboratorio de Mamíferos Acuáticos, Departamento de Sistemática y Ecología Acuática El Colegio de Frontera Sur Chetumal México; ^6^ Fundación Internacional para la Naturaleza y la Sustentabilidad Chetumal México

**Keywords:** estradiol, hormonal profile, immunoassay, monitoring, progesterone, reproductive endocrinology

## Abstract

Reproductive endocrinology plays a crucial role in the management of endangered species. Using non‐invasive techniques, the complex nature of their reproductive biology can be better understood. This study aimed to determine hormonal concentrations of progesterone (P4) and estradiol (E2) in female Greater Caribbean manatees (*Trichechus manatus manatus*) under human care and to establish whether there are differences in hormone concentrations between sexually mature and sexually immature females based on fecal analysis. The assessment of steroid metabolites was performed using the enzyme‐linked immunosorbent assay (ELISA) technique with a commercial DetectX kit. The results indicated reference values for progesterone (P4) ranging from 109.9 to 334.3 ng/g feces and for estradiol (E2) ranging from 12.2 to 90.9 ng/g feces. These results suggest an inversely proportional relationship between the two hormones, demonstrating a complex interaction in the estrous cycle of manatees. The findings also indicated no evidence that individual hormonal levels are correlated (Spearman correlation test: *ρ* = 0.122, *p* = 0.254; and Kendall correlation test: *τ* = 0.084, *p* = 0.245), although higher progesterone levels were associated with lower estradiol concentrations and vice versa. Similarly, no significant correlation was observed between the hormone concentrations of sexually mature and sexually immature females (Skillings–Mack test: P₄, *p* = 0.103, df = 34, 10,000 repetitions; E₂, *p* = 0.276, df = 34, 10,000 repetitions). Longer monitoring periods, along with vaginal cytology and ultrasound evaluations, are recommended.

## Graphical abstract

This study established the first fecal hormonal reference values (P4/E2) for the Greater Caribbean manatee. Using non‐invasive ELISA, the authors found no significant differences between mature and immature females, highlighting that long‐term monitoring and ultrasounds are essential to fully understand their complex reproductive cycles.

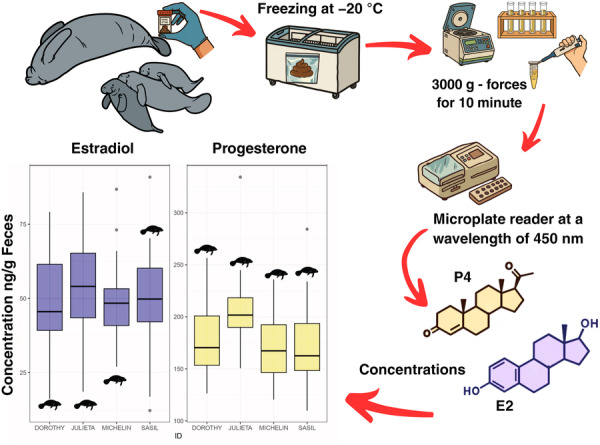

## 1 Introduction

Knowledge of reproductive cycles in marine mammals has been based on the sampling of a limited number of individuals and species (e.g., Atkinson et al. 2023; Guinn et al. 2024; Olsen et al. 2025; Smoll et al. 2025). Consequently, the available information has served as a baseline reference for numerous studies. Physiological aspects in marine mammals—including hormonal profiles and estrous cycle duration—can vary greatly even within the same taxonomic group (Katsumata 2010; Pomeroy 2011). For instance, estrous cycle lengths vary within species belonging to the family Delphinidae: ~31 days in Pacific white‐sided dolphins *Lagenorhynchus obliquidens* (Robeck et al. 2009), ~36 days in bottlenose dolphins *Tursiops truncatus* (Robeck et al. 2005), and ~34 days in belugas *Delphinapterus leucas* (Steinman et al. 2012). Moreover, these variations are often influenced by external or internal factors such as noise and chemical pollution (e.g., Murphy et al. 2010), prey availability (e.g., Nabi et al. 2018; Ward et al. 2009), photoperiod (e.g., Tomita et al. 2011), and nutritional status (e.g., IJsseldijk et al. 2021), which may alter reproductive investment or even induce anestrus. Therefore, it is essential to deepen research on these reproductive processes and the factors that may alter them (Pomeroy 2011).

In the order Sirenia, reproductive physiology has been studied using various monitoring strategies. Horikoshi (2004) reported progesterone (P4) concentrations of 102, 66, and 5.06 ng/g, and estradiol (E2) concentrations of 128.19 and 5.0 ng/g in Florida manatees (*Trichechus manatus latirostris*), using feces as the biological matrix. Wakai et al. (2002) reported P4 concentrations of 1.94 and 0.01 ng/mgCr and E2 concentrations of 0.09 and 23.7 ng/mgCr in dugongs (*Dugong dugon*) using urine as the biological matrix. Amaral et al. (2013) used saliva as the matrix in Amazonian manatees (*Trichechus inunguis*), obtaining P4 concentrations of 62.89 and 2.84 pg/mL and E2 concentrations of 19.64 and 4.26 pg/mL. Using the same approach, the estrous cycle length was determined to range from 28 to 42 days in *T. manatus latirostris* (Horikoshi 2004; Larkin 2000), from 42 to 48 days in *T. inunguis* (Amaral et al. 2013), and 53.6 days in *D. dugon* (Wakai et al. 2002). However, for some taxa, such as the African manatee *T. senegalensis* or the Greater Caribbean manatee subspecies *T. m. manatus*, much of this information remains unknown (Brammer‐Robbins et al. 2024).

The monitoring of steroid hormones has proven to be an effective tool for understanding the biology of various species (Amaral 2010). Immunoassays using specific antibodies for estrogens or antibodies against total unconjugated estrogens are particularly reliable, especially in species where the placenta serves as a significant source of estrogens (Schwarzenberger et al. 1996). Traditionally, plasma or serum has been the most used matrix for this purpose. However, blood sampling in aquatic mammals is a highly invasive and stressful procedure, particularly in endocrine studies that require daily collections to identify reproductive events such as ovulation (Whitten et al. 1998). In individuals without medical training, sampling involves restraint and removal of the animal from the water, which not only increases the risk of physiological stress but also limits its feasibility under ethical management conditions or in wild individuals. This would introduce an immeasurable bias, as some animals would be trained for sampling while others would not. Furthermore, repeated blood sampling may lead to hematological complications such as phlebitis, decreased hematocrit, or even venous collapse in long‐term studies (Whitten et al. 1998).

Considering that ovarian cycle analysis requires continuous and prolonged sample series, and that the hormonal profiles of this subspecies have not yet been described, fecal samples represent the most suitable matrix for endocrine monitoring. This type of sampling enables non‐invasive monitoring, minimizes behavioral impact, and facilitates systematic data collection over extended time periods. Once baseline hormonal patterns are established, less frequent sampling schemes may become feasible; however, during initial stages, continuous fecal monitoring is essential to establish reliable physiological references for the estrous cycle.

Therefore, non‐invasive hormonal monitoring through fecal analysis, using immunoenzymatic techniques, such as Enzyme‐Linked Immunosorbent Assay (ELISA), represents the most suitable alternative for studying hormonal fluctuations. This approach is essential for predicting hormonal peaks that trigger reproductive activity and for identifying environmental and anthropogenic factors that may cause stress and influence fertility in wild populations (Wilson et al. 2011). Additionally, characterization of steroids is crucial for estimating reproductive competence, detecting individuals with fertility issues, and evaluating the need for interventions through assisted reproductive techniques (Robeck et al. 1994). The analysis of urinary and/or fecal steroid metabolites can be potentially useful for evaluating the estrous cycle (e.g., Horikoshi 2004; Larkin 2000), gestation (e.g., Burgess et al. 2012), sexual maturity (e.g., Lanyon and Burgess 2014), reproductive behavior, and seasonal patterns in sirenians.

The Greater Caribbean manatee (*T. manatus manatus*) is classified as Endangered (EN) by the International Union for Conservation of Nature (IUCN) Red List, with a declining population (Morales‐Vela et al. 2024). Its vulnerability in Mexico is attributed to factors such as low reproductive rate, limited genetic variability, habitat fragmentation, and the effects of climate change (Secretaría de Medio Ambiente y Recursos Naturales [SEMARNAT] 2020). Hence, understanding the reproductive endocrinology of the Endangered Greater Caribbean manatee is essential for guiding policy and management decisions, ultimately enhancing protection efforts.

However, research in this field has been limited due, among others, to the low efficiency of conventional reproductive monitoring tools (Burgess et al. 2012). In this context, standardizing the technique in manatees under human care represents a crucial preliminary step before its application to wild populations, as it allows identification of the most suitable matrix for field use and validation of both analytical and sampling procedures. The physiological information derived from these baseline studies is essential for interpreting hormonal profiles in free‐ranging individuals and, ultimately, can contribute to assessing the reproductive potential and overall health status of wild populations (Whitten et al. 1998).

This study proposes the use of non‐invasive techniques for monitoring steroid hormones through immunoenzymatic methods (ELISA) applied to the Greater Caribbean manatee (*Trichechus manatus manatus*). The objective was to determine progesterone (P4) and estradiol (E2) concentrations in female Greater Caribbean manatees under human care, and to establish whether there are differences in hormone concentrations between sexually mature and sexually immature females based on fecal analysis.

## 2 Methods

### 2.1 Sample Collection

This study was conducted at The Dolphin Company Group's dolphinarium, located in Puerto Aventuras, Quintana Roo, Mexico. Four female manatees, two sexually mature and two sexually immature, aged between 2 and 21 years old at the time of the study, were monitored. Details on their life and reproductive history are shown in Table 1. They cohabited with a male until 2020, but since then, the individuals have remained in an exclusively female group. Their diet consisted of papaya, banana, lettuce, spinach, and hydroponic corn sprouts, and all individuals were in good health according to continuous veterinary monitoring. The individuals received regular veterinary assessments: a Daily Presentation Evaluation was performed every day, including observation of behavior, attitude, appetite, body condition, skin, eyes, and oral cavity. In addition, blood samples were collected two to four times per year, or more frequently if clinical monitoring was required for a specific condition.

**Table 1:** Biological and reproductive history of the four female Greater Caribbean manatees (*Trichechus manatus manatus*) included in this study.

ID	Origin	Date of admission/birth	Age	Reproductive status	Number of births	Number of fecal samples
Julieta	Wild‐born	1/05/01	21	Sexually mature	8	20
Dorothy	Wild‐born	9/05/03	15	Sexually mature	1	28
Michelin	Born at Dolphin Discovery	17/05/18	4	Sexually immature	0	16
Sasil	Born at Dolphin Discovery	10/12/20	2	Sexually immature	0	24

**Figure 1:**
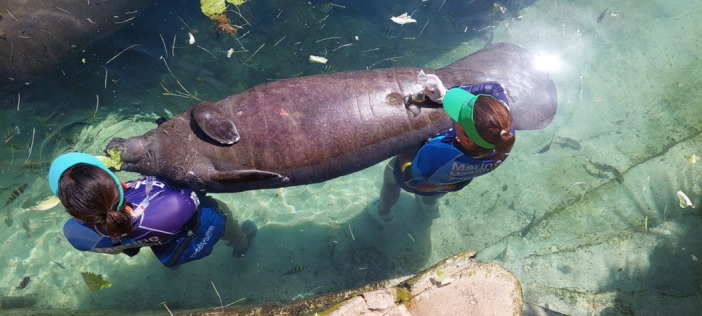
Fecal sample collection in female Greater Caribbean manatees (*Trichechus manatus manatus*) under professional care, through operant conditioning. Photo: V. Bermúdez‐Cardona.

Sampling was conducted over 45 days, taking as reference the duration of the estrous cycle of the Florida manatee (28–42 days), with the purpose of covering at least one complete cycle. The sampling period extended from October 15 to November 30, 2022. Fecal material was collected between 10:00 and 12:00 h using operant conditioning to encourage voluntary sample submission (Figure 1), a technique recommended for marine mammals when husbandry conditions allow (e.g., Bai et al. 2021; Biancani et al. 2009). Approximately 5 g of feces were manually obtained per individual, collected directly from the anus of each female using latex gloves. A total of 88 samples were gathered, although the number varied among individuals: 20 from Julieta, 28 from Dorothy, 16 from Michelin, and 24 from Sasil. This variation occurred because on certain days, the animals were not cooperative or simply did not defecate during the sampling period. The samples were placed in Whirl‐Pak bags and subsequently stored at −20°C at the Dolphin Discovery laboratory in Puerto Aventuras until processing, which was carried out 56 days after collection.

For environmental temperature monitoring, the facility was equipped with HOBO Pendant MX Temp (MX2201) thermometers. These devices, designed to withstand submersion up to 30 m (approximately 100 ft), are equipped with Bluetooth Low Energy technology that allows wireless data transfer. They have an accuracy of ±0.5°C (±0.9°F) and were programmed to record temperature continuously throughout the year. Measurements were taken every 6 h (at 05:33, 11:33, 17:33, and 23:33 h).

### 2.2 Sample Analysis

Sample analyses were carried out at the Laboratory of Reproduction and Endocrinology of the General Directorate of Zoos and Wildlife Conservation in Mexico City. Steroid quantification was performed using competitive ELISA with DetectX kits from Arbor Assays (catalog numbers K025‐H1/H5 and K030‐H1/H5), specifically designed for the quantitative measurement of progesterone (P4) and estradiol (E2) in dried fecal samples. Arbor Assays is an employee‐owned company based in Ann Arbor, Michigan (USA), dedicated to the development of high‐quality detection and immunoassay kits. These kits contain antibodies specifically developed for the detection of P4 and E2 in urine and fecal samples and are not recommended for use with serum, plasma, or saliva. According to the manufacturer, P4 and E2 molecules are structurally identical across species, allowing their application in different taxa. These kits have also been previously used for fecal steroid quantification in wild white‐faced capuchins *Cebus imitator* (Beehner et al. 2022), the California sea lion, *Zalophus californianus* (Morales Pérez 2020), the blue whale, *Balaenoptera musculus* (Valenzuela‐Molina et al. 2018), and the female bowhead whale, *Balaena mysticetus* (Lysiak et al. 2023).

For the preparation of the controls, known values were used to ensure the reliability of the assays. For the estradiol assay, two diluted standards from the calibration curve were employed: 5000 and 78.125 pg/mL. Additionally, an internal standard of 500 pg/mL was prepared from a previously characterized sample. For the progesterone assay, the standards were used directly, selecting the 1600 and 100 pg/mL points, and an internal standard of 1200 pg/mL was included. These internal controls served as positive controls, allowing the evaluation of intra‐ and inter‐assay variation. The phosphate‐buffered saline solution (0.1 M, pH 7.2) used for dilutions was also employed to determine non‐specific binding, functioning as the negative control.

Before analytical processing, serial dilutions were performed to determine the optimal working concentration, as the samples initially showed high hormone levels. The dilutions tested were 1/2, 1/5, 1/10, 1/50, and 1/100. For progesterone, the appropriate dilution was 1/10, whereas for estradiol it was 1/5. Samples that deviated from the 50% binding point but remained within the detectable range yielded consistent values, confirming the reliability of the selected dilutions.

The extraction of steroid metabolites was conducted by adapting the protocols of Wasser et al. (1994), Brown et al. (2004), and Sandoval‐Zarate and Sánchez‐Espinoza (2024). The procedure involved several sequential steps: recording the fresh sample weight, dehydrating the feces in an oven at 70 ± 5°C for approximately 4 h, and processing the material in sterile borosilicate aliquots. Subsequently, 0.3 g of dried feces were placed in each tube, followed by the addition of 3 mL of 90% methanol (1:10 ratio). The tubes were sealed with Parafilm, vortexed for 1 min, and then placed on a tube rotator for 12 h. Afterward, the samples were centrifuged for 10 min at 3000 g‐ forces (1330 × *g*), and the debris‐free supernatant was transferred to microtubes and stored at −20°C until ELISA analysis.

Fecal progesterone (P4) metabolites were quantified using the DetectX Progesterone Enzyme Immunoassay Kit (Arbor Assays; catalog no. K025). Samples were analyzed at a 1:10 dilution, and the standard calibration curve consisted of seven points ranging from 50 to 3200 pg/mL. Assays were conducted on clear 96‐well microtiter plates pre‐coated with mouse monoclonal antibodies specific to progesterone and stored at 4°C until use. All samples were analyzed in duplicate, with 50 µL loaded per well, and the analytical sensitivity of the assay was 47.9 pg/mL. Fecal estradiol (E2) metabolites were measured using the DetectX Estradiol Enzyme Immunoassay Kit (Arbor Assays; catalog no. K030‐H). Samples were analyzed at a 1:5 dilution, with a standard curve comprising five calibration points ranging from 39.06 to 10,000 pg/mL. Assays were performed in clear 96‐well microtiter plates coated with rabbit monoclonal antibodies specific for estradiol and stored at 4°C until use. All samples were analyzed in duplicate (50 µL per well), and the assay sensitivity was 39.6 pg/mL. Analytical validation for both progesterone and estradiol assays included preliminary testing of fecal extracts from each individual to confirm binding percentages close to 50% relative to the standard calibration curves, using the DetectX kits (K025 and K030‐H; Arbor Assays). Analytical accuracy was assessed by calculating intra‐ and inter‐assay coefficients of variation, which were maintained below 15%, consistent with established standards for fecal steroid hormone analyses (Brown et al. 2004).

The ELISAs for steroid hormone quantification were conducted following the manufacturer's instructions. Optical density was quantified at a wavelength of 450 nm using an ELX880 plate reader. The specificity test, according to the manufacturer, is 100% for progesterone, 3β‐Hydroxy‐progesterone: 172%, 3*α*‐Hydroxy‐progesterone: 188%, 11β‐Hydroxy‐progesterone: 2.7%, 11*α*‐Hydroxy‐progesterone: 147%, 5*α*‐Dihydroprogesterone: 7.0%, Pregnenolone: 5.9%. For estradiol: 17β‐Estradiol: 100%, Estrone: 0.78%, 17*α*‐Estradiol: 0.22%, 17*α*‐Ethinylestradiol: 0.11%.

### 2.3 Data Analysis

Statistical analyses were conducted using R software version 4.3.0. Since the data obtained were non‐parametric, correlations were estimated using Spearman and Kendall tests. To compare hormone concentrations among individuals in different reproductive states, a random subsampling was performed based on the minimum number of observations corresponding to the individual Michelin (*n* = 16), repeating this procedure 100 times. Additionally, to examine differences in progesterone and estradiol concentrations, the Skillings‐Mack statistical method was applied. This is a non‐parametric extension of the Friedman test, specifically designed to handle incomplete block design data (Skillings and Mack 1981).

## 3 Results

In this study, the intra‐assay variation for P4 averaged 9.8%, while for E2 it was 10.75%. The inter‐assay variation was 15.5% for P4 and 11.3% for E2. The minimum recorded P4 concentrations were 109.9 ng/g feces, whereas the maximum reached was 334.3 ng/g feces. For E2, the minimum observed concentration was 12.2 ng/g feces, with a maximum of 90.9 ng/g feces (Table 2). Spearman's correlation test (*ρ* = 0.122, *p* = 0.254) and Kendall's test (*τ* = 0.084, *p* = 0.245) suggest that there is no significant correlation between P4 and E2 concentrations among the individuals analyzed. Likewise, no significant correlation was observed between hormone concentrations in sexually immature versus sexually mature females. The correlation results (Spearman's *ρ* and Kendall's *τ*) did not indicate evidence of a relationship between hormonal levels at the individual level. However, an inverse pattern was observed, in which higher P4 levels corresponded to lower E2 concentrations and vice versa.

**Table 2:** Fecal concentrations (ng/g feces) of progesterone (P4) and estradiol (E2), including the average, maximum, minimum, and standard deviation values in sexually mature (Julieta and Dorothy) and sexually immature (Michelin and Sasil) female Greater Caribbean manatees (*Trichechus manatus manatus*) under professional care.

	Fecal progesterone – P4				Fecal estradiol – E2			
ID	Average	Max	Min	SD	Average	Max	Min	SD
Julieta	205.6	334.3	150.6	39.2	53.2	85.2	18.6	15.9
Dorothy	175.7	256.6	126.4	32.9	48.1	79.2	16.2	16.6
Michelin	170	236	120.4	33.7	49.9	86.8	27	15.6
Sasil	174.2	284.3	109.9	40.8	50.0	90.9	12.2	17.4

**Figure 2:**
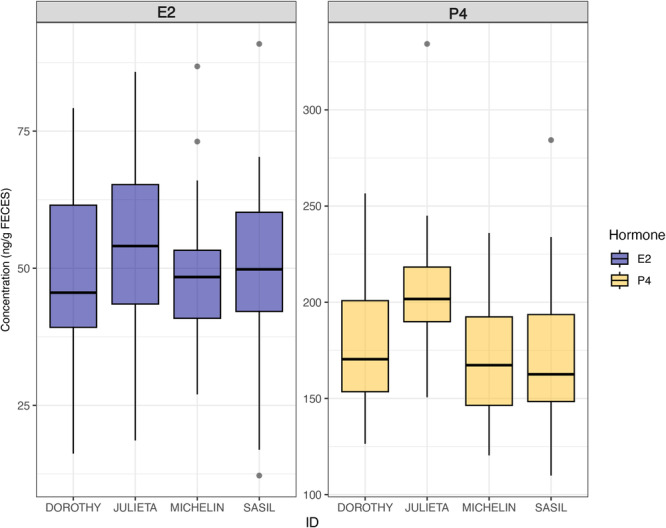
Box plots of fecal estradiol (E2) and progesterone (P4) concentrations (ng/g feces) measured in four female Greater Caribbean manatees (*Trichechus manatus manatus*) under professional care.

**Figure 3:**
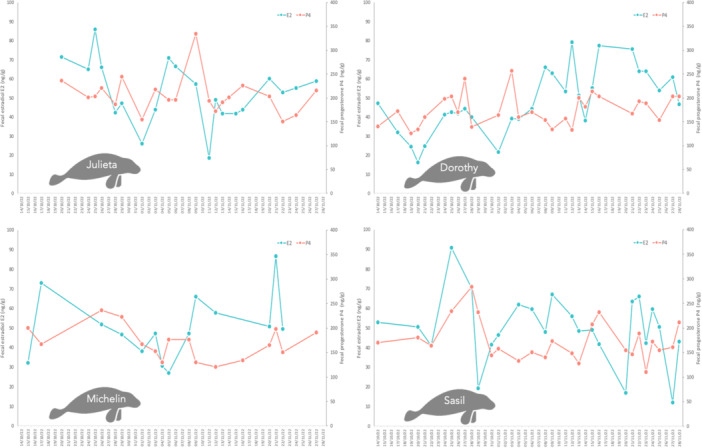
Temporal patterns of fecal estradiol (E2) and progesterone (P4) concentrations in sexually mature (Julieta and Dorothy) and sexually immature (Michelin and Sasil) female Greater Caribbean manatees (*Trichechus manatus manatus*) under professional care.

The results of the Skillings–Mack test for P4 (*p* = 0.103123, df = 34, with 10,000 repetitions) and E2 (*p* = 0.276130, df = 34, with 10,000 repetitions) indicate that steroid concentrations did not differ significantly among the evaluated individuals, as shown in Table 3. This is visually represented in Figure 2. In the graphical representation of hormonal concentrations (Figure 3), an estrogenic phase inversely proportional to the progestational phase can be observed.

**Table 3:** Pairwise comparison of hormonal correlations (*ρ* = Spearman and *τ* = Kendall) between sexually immature (Sasil and Michelin) and sexually mature (Dorothy and Julieta) females, Greater Caribbean manatees (*Trichechus manatus manatus*) under professional care.

ID	Fecal progesterone – P4		Fecal estradiol – E2	
	Sasil	Michelin	Sasil	Michelin
Dorothy	*ρ* = 0.001 ± 0.253	*ρ* = −0.002 ± 0.284	*ρ* = 0.041 ± 0.256	*ρ* = −0.010 ± 0.296
	(0.526 ± 0.300)	(0.488 ± 0.307)	(0.493 ± 0.287)	(0.469 ± 0.317)
	*τ* = −0.002 ± 0.188	*τ* = 0.002 ± 0.210	*τ* = 0.032 ± 0.185	*τ* = −0.006 ± 0.207
	0.508 ± 0.298	(0.482 ± 0.308)	(0.476 ± 0.292)	(0.472 ± 0.310)
Julieta	*ρ* = 0.012 ± 0.294	*ρ* = 0.008 ± 0.244	*ρ* = 0.038 ± 0.268	*ρ* = −0.028 ± 0.289
	(0.464 ± 0.306)	(0.526 ± 0.292)	(0.476 ± 0.291)	(0.495 ± 0.315)
	*τ* = 0.008 ± 0.210	*τ* = 0.003 ± 0.179	*τ* = 0.026 ± 0.193	*τ* = −0.021 ± 0.211
	(0.468 ± 0.309)	(0.514 ± 0.287)	(0.475 ± 0.290)	(0.507 ± 0.326)

*Note:* The estimator values (*ρ* and *τ*) correspond to the mean ± standard deviation of 100 subsampling replicates, using as reference the minimum number of data points (*n* = 16), equivalent to the total number of samples obtained for Michelin.

## 4 Discussion

In this study, for the first time, endocrine evaluation through fecal sample analysis was successfully applied to female Greater Caribbean manatees under professional care. This methodological advancement provides an essential baseline for future research on the reproductive endocrinology of the subspecies and supports the development of non‐invasive endocrinological tools for conservation management of Greater Caribbean manatees under human care. Furthermore, these findings establish a reference framework that can guide the design and interpretation of future studies in wild populations, acknowledging that additional validation will be required to account for environmental and dietary differences.

The ELISA technique, using a DetectX commercial kit, proved to be efficient, as indicated by previous research (Amaral 2010; Burgess et al. 2012, 2013; Horikoshi 2004; Larkin 2000). The obtained hormone concentrations were highly specific, establishing reference values for estradiol and progesterone for the subspecies. Although full biological validation (e.g., pharmacological challenges or comparison with circulating hormone profiles) was not feasible due to ethical and logistical constraints associated with working with an endangered species under human care, we implemented several analytical validation steps to ensure the assays were appropriate for Greater Caribbean manatee fecal samples, following the procedures described by Larkin (2000) and Horikoshi (2004). Specifically, we conducted parallelism tests using serial dilutions of pooled manatee fecal extracts, which demonstrated parallel displacement curves relative to the standard curve, indicating that the assay antibodies appropriately recognize hormone metabolites present in manatee feces. In addition, recovery tests were performed by spiking fecal extracts with known amounts of hormone standards, yielding acceptable recovery rates within the range reported for validated fecal steroid assays. Assay precision was also assessed through intra‐ and inter‐assay coefficients of variation, which fell within acceptable limits for endocrine studies. While the analytical validation supports the reliability of the measurements, further biological validation (e.g., longitudinal monitoring across known reproductive events, ultrasound confirmation, or comparison with serum hormones) is warranted and forms part of our recommendations for future research.

A major methodological improvement was the direct collection of fecal samples from each individual, rather than from water, which reduced the risk of mixing samples between animals and increased the precision of individual identification. However, because this procedure depended on the animals' voluntary approach, sample collection was not forced when individuals did not cooperate. Consequently, the number of samples obtained varied among individuals, highlighting the need for longer sampling periods in future studies to achieve a comprehensive characterization of the estrous cycle.

Analysis of P4 and E2 concentrations did not allow identification of physiological differences between sexually mature and sexually immature individuals. This finding is consistent with the reports of Larkin (2000) and Horikoshi (2004), who also found high variability in hormonal concentrations in female Florida manatees at different ages. In some cases, the hormone levels were so low that they were not detectable using radioimmunoassay. Despite the lack of hormonal differences between reproductive states, it is important to mention that Sasil, a 2‐year‐old prepubertal female, was observed to have fluctuating E2 levels, generally elevated compared to adult females, suggesting a possible onset of puberty. This result aligns with the findings of Burgess et al. (2013), where a progressive increase in E2 levels was documented in a female dugong of the same age. Overall, the hormonal concentrations were similar among individuals, and this lack of significant variation could be influenced by intrinsic factors such as genetic and physiological characteristics of each individual. A larger sample size of female manatees is recommended to better understand inter‐individual differences.

Likewise, studied individuals exhibited similar hormonal patterns. We were unable to determine whether they were completing or initiating a new estrous cycle through the endocrinological analysis, but it is possible that the females were in an anestrous phase during the sampling period, as no evident signs of estrus were observed. This condition could be influenced by external factors, such as the fact that this social group was made exclusively of females, or the season in which the sampling took place. This highlights the importance of considering environmental factors and management conditions in the reproductive physiology of sirenians, as well as individual variability in sexual maturation within this group of aquatic mammals.

Based on the data obtained, the presence of an estrogenic phase as well as a progestational phase was identified, characterized by an inverse relationship between both hormones. This suggests a complex regulatory interaction between progesterone and estradiol, indicating an intrinsic control mechanism within the estrous cycle. Larkin (2000) and Horikoshi (2004) documented increases in estradiol levels in most of the females analyzed, observing that one‐third of these increases preceded rises in progesterone by at least 2 weeks. Amaral et al. (2013) also reported that estradiol peaks coincided with baseline progesterone levels in the Amazonian manatee. The authors suggested that these hormonal peaks may play a key role in reproductive behavior and male attraction.

Previous studies have documented variations in estrous cycle length among different sirenian species, even though they belong to the same family. Reported cycle durations range from 28 to 42 days in *T. manatus latirostris* (Horikoshi 2004; Larkin 2000), 53.6 days in *D. dugon* (Wakai et al. 2002), and an average of 44.67 days in *T. inunguis* (Amaral et al. 2013). These differences suggest that both cycle length and hormonal concentrations may vary among species within this group. In the case of the Greater Caribbean manatee, no prior references exist regarding the duration of reproductive cycles. Thus, it is inferred that they may be similar to those reported for other sirenians, approximately 40 days. Under this assumption, the sampling period implemented in this study may have been shorter than the full reproductive cycle of the subspecies. Among the individuals analyzed, only Dorothy showed a clear trend in hormonal concentrations. Dorothy, an adult sexually mature female, was the individual who provided the largest number of samples (28), allowing for a more consistent hormonal pattern to be identified. As shown in Figure 3, her profile exhibits a distinct estrogenic phase, a progestational phase, and defined variations in fecal steroid concentrations. It is also important to note that two of the females evaluated were classified as sexually immature. Both the limited continuity in sample collection and the age of some individuals may have contributed to the absence of evident hormonal increases in all cases.

In the present study, the facility maintained continuous temperature monitoring, with an average water temperature of 29°C throughout the evaluation period, and the females had permanent access to freshwater. Therefore, neither temperature nor freshwater availability appears to have influenced the estrous cycle of the individuals analyzed, and the hypothesis of reproductive seasonality in this subspecies lacks solid supporting evidence. The potential influence of seasonality on the reproduction of Greater Caribbean manatees remains uncertain. Reproductive seasonality in this subspecies is not evident, possibly because water temperatures in the tropics rarely fall below 25°C during winter. However, manatee movements may be influenced more by the availability of freshwater than by thermal fluctuations (Self‐Sullivan et al. 2003). Given the absence of reproductive data for Greater Caribbean manatees, both under professional care and in the wild, extensive research is necessary to identify patterns of reproductive seasonality and the factors influencing mating behavior (Brammer‐Robbins et al. 2024).

A longer monitoring period is recommended, with sample collection every other day for at least 8 months, to more accurately identify the number of progesterone (P4) and estradiol (E2) peaks, determine cycle length, and better assess the potential influence of seasonality in this subspecies. Furthermore, these studies should be complemented with vaginal cytology analyses, ultrasonography, and behavioral assessments. Our results represent a starting point for establishing baseline values of fecal steroid concentrations in Greater Caribbean manatees, thus providing a basis for future studies on the reproductive aspects of the subspecies.

## Author Contributions

**Vanessa Bermúdez‐Cardona:** conceptualization, methodology, investigation, data curation, formal analysis, validation, visualization, writing – original draft, writing – review and editing. **Roberto Sánchez‐Okrucky:** conceptualization, resources, methodology, investigation, validation, writing – review and editing. **José A. Sandoval‐Zárate:** conceptualization, resources, methodology, investigation, supervision, validation, writing – review and editing. **Nataly Castelblanco‐Martínez:** conceptualization, resources, methodology, investigation, supervision, validation, visualization, writing – review and editing.

All authors contributed substantially and were fundamental to the development of this study. They participated in the conception and design of the research, data collection and analysis, interpretation of results, and preparation and critical revision of the manuscript. All authors approved the final version of the manuscript and agree to be accountable for all aspects of the work.

## Ethics Statement

All procedures were conducted in compliance with the Mexican standard NOM‐062‐ZOO‐1999 regarding animal ethics and welfare, the General Wildlife Law, and the Federal Animal Health Law. AI‐assisted tools were employed exclusively for linguistic refinement and clarity. The intellectual content, including all ideas, analyses, and conclusions, was conceived and verified by the authors, who take full responsibility for the manuscript.

## Conflicts of Interest

The authors declare no conflicts of interest.

## Data Availability

All data presented in this article are publicly available, as they form part of the research conducted for a master's thesis by the author and co‐authors.
